# Potential of accelerometers to remotely early detect bovine ephemeral fever in cattle using pattern mining

**DOI:** 10.1093/tas/txaf008

**Published:** 2025-01-25

**Authors:** Ly Ly Trieu, Derek W Bailey, Huiping Cao, Tran Cao Son, Justin Macor, Mark G Trotter, Lauren O’Connor, Colin T Tobin

**Affiliations:** Department of Computer Science, New Mexico State University, Las Cruces, NM 88003, USA; Department of Animal and Ranges Sciences, New Mexico State University, Las Cruces, NM 88003, USA; Institute for Future Farming Systems, School of Health, Medical, and Applied Sciences, CQUniversity, Rockhampton, QLD 4700, Australia; Department of Computer Science, New Mexico State University, Las Cruces, NM 88003, USA; Department of Computer Science, New Mexico State University, Las Cruces, NM 88003, USA; Institute for Future Farming Systems, School of Health, Medical, and Applied Sciences, CQUniversity, Rockhampton, QLD 4700, Australia; Institute for Future Farming Systems, School of Health, Medical, and Applied Sciences, CQUniversity, Rockhampton, QLD 4700, Australia; Institute for Future Farming Systems, School of Health, Medical, and Applied Sciences, CQUniversity, Rockhampton, QLD 4700, Australia; Carrington Research Extension Center, North Dakota State University, Carrington, ND 58421, USA

**Keywords:** accelerometer, bovine ephemeral fever, on-animal sensor, pattern mining, unsupervised machine learning

## Abstract

Bovine Ephemeral Fever (BEF), caused by an arthropod-borne rhabdovirus, is widespread in tropical and subtropical regions. It affects cattle with symptoms of fever, lameness, inappetence and in some situations can result in mortality. The goal of this study is to determine if accelerometer data can be used to identify the behavior patterns that occur when cattle become ill from BEF. Eight heifers in a separate experiment were monitored with 3-axis accelerometers sensors. Movement variation (MV) was calculated from accelerometer data (25 Hz) using 1-min epochs and then averaged hourly. Two different approaches, cosine similarity (CS) and deviation from previous behavioral patterns, were developed to autonomously detect patterns and recognize the onset of sickness in cattle using accelerometer data. Analyses show that one heifer had behavioral changes one day before the manager observed BEF, and another heifer had behavioral changes on the same day the manager observed BEF. The other six heifers did not display any BEF symptoms. To validate the efficacy of our analytical approaches, we employed them on a separate commercial herd of 73 cows where 4 of the 27 monitored cows were observed with BEF symptoms. Predictions were either on the day or even the day prior to the manager’s observation and diagnosis. There were likely no false positives in the first or second trials using the deviation algorithm with sum_deviation formula, but there were several false positives with the other algorithms. These case studies demonstrate the potential of accelerometer data to autonomously detect disease onset, in some cases before it was apparent to the human observer. However, more research is needed to minimize false positives that may occur from other similar diseases, abnormal weather events or cyclical changes in behavior such as estrus is required.

## Teaser

Two different approaches, cosine similarity and deviation, have the potential to identify changes the behavior pattern of cattle when they become ill from Bovine Ephemeral Fever using accelerometer data. Remote monitoring of livestock health with accelerometers and algorithms such as those described in this study could reduce labor costs and reduce time to treatment in extensive pastures.

## INTRODUCTION

Determining when cattle become ill on rangelands is difficult, because it is labor intensive and often impractical to observe cattle. Recently, real and near real-time sensors are being developed that can remotely monitor livestock (Bailey et al., 2021; Tobin et al., 2022). Accelerometers have been used in diverse environments and applications to remotely monitor livestock activity (Fogarty et al., 2020; Pavlovic et al., 2021). Accelerometers are capable of detecting illnesses, such as Perennial Ryegrass Staggers (Trieu et al., 2022), consumption of mold-contaminated feed (Gurule et al., 2022), internal parasites (Ikurior et al., 2020; Fogarty et al., 2023), and adverse parturition outcomes (Fogarty et al., 2022). Rapid technological advancements such as the Internet of Things, LoRaWAN (Tzounis et al., 2017; Suji Prasad et al., 2021) and satellite communication platforms have facilitated the real-time reception of sensor data, promising the capability to remotely and autonomously provide information to rangeland livestock managers.

Currently, farmers and ranchers must observe livestock to determine if they are ill (Bailey, 2016). This process is often time-consuming, especially for livestock grazing extensive rangelands, and often delays treatment or in some cases prohibits disease management altogether. On-animal sensors have the potential to help alleviate this problem by remotely monitoring changes in animal behavior, which often occurs when animals begin to demonstrate disease related behavior (Bailey et al., 2018; Tobin et al., 2022).

Cattle in tropical and subtropical regions of the world such as Australia, Japan, China, and Africa are commonly exposed to bovine ephemeral fever (BEF) or 3-d sickness (Nandi and Negi, 1999; Walker and Klement, 2015). Bovine ephemeral fever is an insect-transmitted, noncontagious, and viral infection of cattle (Bos taurus, Bos indicus, and Bos javanicus) and water buffalo (Bubalus bubalis). Caused by a rhabdovirus and transmitted by flying, biting insects, BEF has different severity of clinical signs and occur suddenly including biphasic to polyphasic fever (41–42 °C), lameness, inappetence, listlessness, lachrymation, serous nasal discharge, drooling, joint pain and stiffness (Nandi and Negi, 1999). In most of the cases, milk production is lower than normal levels after recovery. Due to the loss of milk production and cattle body condition as well as occasional mortality, BEF can have a major economic impact (Nandi and Negi, 1999; Walker, 2005).

This paper aims to demonstrate a “proof of concept study” to evaluate the potential of detecting and identifying changes in cattle activity that occur when they become ill with BEF using accelerometers and pattern mining algorithms. Traditionally, to detect sickness in general, most applications rely on a supervised classification method (Bishop and Nasrabadi, 2006), where the goal is to assign an input to a class (sick or healthy) based on patterns learned from labeled data. In contrast, our research combines unsupervised methods (pattern mining in particular) in data mining (Han et al., 2011) to identify sickness. Unlike supervised methods, unsupervised methods are applied to unlabeled data to discover patterns and insights of the data. Pattern mining in data mining is used to detect changes in behavior that indicate illness, which is a decrease in activity. Generally, this pattern mining method identifies illnesses by detecting a decline in activity, which signals that the animal may be unwell; therefore, it is not limited to BEF alone. In our case, we know the cattle became ill with BEF based on observations and blood tests. To detect the changes and identify the patterns, we apply two unsupervised approaches, cosine similarity (CS) and deviation from previous behavioral patterns using accelerometer data from two studies where cattle became ill with BEF. Cosine similarity method is widely applied in various computer science applications such as text mining and data mining (Tan et al., 2016); however, its use is not well recognized in the agriculture field. Deviation method is proposed and developed in this work as an alternative pattern mining method to CS. We hypothesize that these algorithms, CS and deviation, have the capacity to identify changes in behavior that occur when heifers become ill with BEF. We also determine if these algorithms initially evaluated with heifers are effective in a separate large scale commercial herd of cows.

## MATERIAL AND METHODS

### Study Sites

The first trial was conducted at the Central Queensland Innovation & Research Precinct (150°30′E, 23°19′S, elevation 40 m), Rockhampton, Queensland, Australia from 24 August to 2 October 2016. The size of paddocks varied from 1–10 ha. Additional information on the study site and environment can be found in (Williams et al., 2017).

The second trial utilized data from a 3-mo study on dystocia in cows located on a commercial cattle operation in the Kilcummin region of Central Queensland approximately 40km north of Clermont, Queensland, Australia. The longitude and latitude of the middle of the paddock are 147˚31.04’E, and 22˚29.74’S. The 191-ha paddock is characterized by a concentration of trees in its northwest quadrant, with the pasture predominantly composed of Buffel grass (*Cenchrus ciliaris*) on brigalow black soil.

The research protocol of the first trial in Rockhampton was approved by the CQUniversity Animal Ethics Committee with the approval number 20119. The second study was approved by the CQUniversity Animal Ethics Committee with the approval number 22424.

The first trial used data from a water drinking experiment, described by (Williams et al., 2017). The eight yearling Brahman and Brahman-cross heifers were grazed in 1–10 ha paddocks in a rotational grazing system. The heifers grazed in the rotational grazing system from 24 August to 2 October 2016. At the beginning of the study, the mean live weight of the heifers was 266 kg (s.d. 27 kg, range 229–303 kg). Eight heifers were managed together and had been run at the study site for the 6 mo prior to the BEF incident. The research used triaxial ± 16 g accelerometers (USB Accelerometer X16-4, Gulf Coast Data Concepts, LLC, Waveland, USA). The accelerometer was put in a waterproof ABS enclosure (AEK GmbH, Frankfurt, Germany) that was fitted in a 50 mm belt webbing collar. The position of accelerometers was under the neck, and it recorded movements of the z-front-to-back, y-side-to-side and x-vertical. Movement data were recorded continuously at a rate of 25 Hz. The life of battery was about 245h. The 16 accelerometers used during the 6 wk of this study were divided into two sets. At the beginning of the experiment, accelerometers were randomly assigned to heifers. Then, the accelerometers attached to each heifer were exchanged every 7 d, such that the first accelerometer was assigned in weeks 1, 3 and 5 and the second accelerometer was assigned in weeks 2, 4 and 6. This exchange occurred in a yard where the heifers were trailed to and from their paddock.

The second trial included 73 cows, all of which were around 2 yr old and yearling-mated heifers. As of October 26, 2021, the average weight of these cows was 445 kg ± 55 sd. All of the cattle were equipped with GPS collars, which operated on a 15-min sample interval. Out of the 73 cattle, 27 cows were additionally outfitted with accelerometer ear tags (AX3 Puck, Axivity Ltd., Newcastle, United Kingdom). Tags were configured with a sample rate of 12.5Hz (12.5 readings/second). The battery life varied, typically lasting less than 3 mo. The devices were first attached to cows on October 26, 2021. However, Trial 2 began on December 8^th^, 2021, and new Axivity sensors were placed on the cows, and the batteries for the GPS devices were replaced. All devices were collected on March 7^th^, 2022, and Trial 2 ended.

### Bovine Ephemeral Fever

During Trial 1, the manager observed the heifers daily to determine their overall health. The manager suspected BEF, because the two heifers were not grazing, depressed, reluctant to move, and had nasal discharge. The BEF blood test was obtained within 24 h after first observing the BEF symptoms. The manager did not observe any signs of illness from the other 6 heifers during Trial 1. Trial 2 used a commercial herd. The manager observed the herd on a daily basis. Similar to Trial 1, the manager observed the cattle’s overall health, and noted cows that were not grazing, reluctant to move, depressed, and had nasal discharge as symptoms for 3-d sickness. A sample of the cows (15 of the 27 cows with accelerometers) were given a BEF blood test at the end of the study (March 7, 2022) when the accelerometers were removed.

## ACCELEROMETER DATA ANALYSES

### Data Preprocessing

In the Rockhampton trial, raw accelerometer data recorded were downloaded from the device and initially partitioned into 1-min epochs. For each heifer, the accelerometer devices were changed every Wednesday; thus, the accelerometer data recorded on Wednesdays were not used in our study. Movement Intensity (MI) and Movement Variation (MV) (Fogarty et al., 2020) were calculated for each 1-min epoch (Table 1). The 1-min epoch accelerometer metrics were then averaged into 1-h periods for pattern mining. Only 1-min epochs without any missing records were used. All epochs used contained at least 1,400 records. The epoch contained missing data was not used. First, the MI and MV metrics were multiplied by 1,000 to emphasize differences when applying deviation approach. Then, the metrics further processed using weekly normalization and smoothing that are described below.

**Table 1.** Two features and the equation are used to calculate values

Feature	Equation
Movement intensity (MI)	$MI=\;\frac{1}{T}\overset{T}{\underset{t=1}{\sum }}\sqrt{x{\left(t\right)}^{2}+y{\left(t\right)}^{2}+z{\left(t\right)}^{2}}$
Movement variation (MV)	$MV=\frac{1}{T}\overset{T}{\underset{t=2}{\sum }}\left(\left|x\left(t-1\right)-x\left(t\right)\right|+\left|y\left(t-1\right)-y\left(t\right)\right|+\;\left|z\left(t-1\right)-z\left(t\right)\right|\right)$

Tobin et al. (2024) found that MV was more effective for detecting changes in behavior than MI. Movement variation is calculated from changes in accelerometer movement rather than the actual that actual values of accelerometer movements used for MI (Table 1), which makes it less sensitive to variation among animals and devices. In addition, our preliminary analyses showed that MV was superior to MI for detecting BEF. For brevity, we report results and outcomes using MV and not MI.

#### Weekly normalization.

Because the accelerometer devices were changed weekly in the first trial, there was the potential for variance in the amplitude among devices placed on the same heifer. Thus, we applied a weekly normalization account for the variation between devices placed on the same heifer. We noticed that the absolute values of the raw accelerometer data and calculated metrics varied by a consistent amount when the accelerometers were changed weekly. Additionally, this was anecdotally observed in Tobin et al. (2020) and Tobin et al. (2024) further documented the differences among accelerometers placed on the same animals. The methodology for this process follows: 1) the mean of first week of accelerometer metrics was the base; 2) deviation of the mean of following weeks was added each of accelerometer metrics for that week. For example, if the first week mean metric value was 1,000 (base) and mean for the 2 wk was 950, 50 was added to every metric value for the 2 wk. The goal was not to change the variance in the metrics, but to adjust the magnitude of the metrics for differences in the absolute values of the accelerometers.

#### Data smoothing.

Variation in cattle diurnal behavior patterns results in noisy data which makes detection of changes in behavior difficult. To mitigate this, we employed a data smoothing process, which involves using the following equation to recalibrate the accelerometer metric at hour t on day D, represented as $D_{t^{\prime }}$:


$$\begin{array}{l} D_{t^{\prime }}=\frac{1}{2*w+1}\;\underset{i=t-w}{\overset{t+w}{\sum }}\;D_{i} \end{array}$$

In the equation mentioned, $D_{i}$ represents an accelerometer metric at hour i on day D. The variable w denotes the window size, which is applied in the formula to retrieve w times the 1-h metric values both before and after hour t. In our research paper, we have set w to 1.

### Pattern Mining of Accelerometer Data

Pattern mining is a type of data analysis in which algorithms are utilized to identify patterns and regularities in data. In this study, we use pattern mining to detect abnormalities in accelerometer data before, during and after cattle become ill with BEF. In typical pattern mining problems, a data instance must be defined. We used a 1-h instance as a compromise between a very large data set with finer temporal scales (e.g., 1 min) and excessively coarse scales (e.g., daily). Sick days were considered abnormal and were defined by the period after BEF diagnosis by a veterinarian and confirmed with a blood test. The 1-h epoch accelerometer metrics are denoted as features or attributes. Each data instance consists of multiple features.

In our study, two different approaches are utilized for pattern mining of BEF, Cosine Similarity and deviation. Cosine Similarity is a well-known similarity measure which has been applied in different machine learning algorithms (Tan et al., 2016) while deviation is our newly proposed and developed method based on statistical variation and outlier analyses. These approaches were used in two periods when the cattle were normally active and not resting or inactive.

#### Active and inactive periods.

To better identify changes in behavior that may be associated with illness, we explored variation against the animal’s normal diurnal activity patterns. This allowed us to identify typical active and non-active periods throughout the day. The procedures used to identify the diurnal behavioral patterns are described below.

Given all values of 1-min epochs of MV for a heifer, M, we calculated a threshold, to distinguish active or inactive periods as: threshold= median(M)−0.5∗ σ (M) where σ(M) is the population standard deviation of M. We subtract 0.5* because our hypothesis prioritizes active time over inactive time, and we aim to capture a longer duration of the heifer being active. A 1-min epoch is considered active if MV ≥ threshold. Otherwise, the 1-min epoch is inactive. A 1-h instance is considered as active if the number of 1-min active epoch is more than the number of 1-min inactive epoch. To identify the active or inactive periods of a heifer throughout the day, we examine the most frequent activity (either active or inactive) within 1-h epochs spanning from 0:00–24:00. Similar to MV, the active or inactive period with regard to MI is computed using the same procedures.

### Active Periods

**Figure 1.**
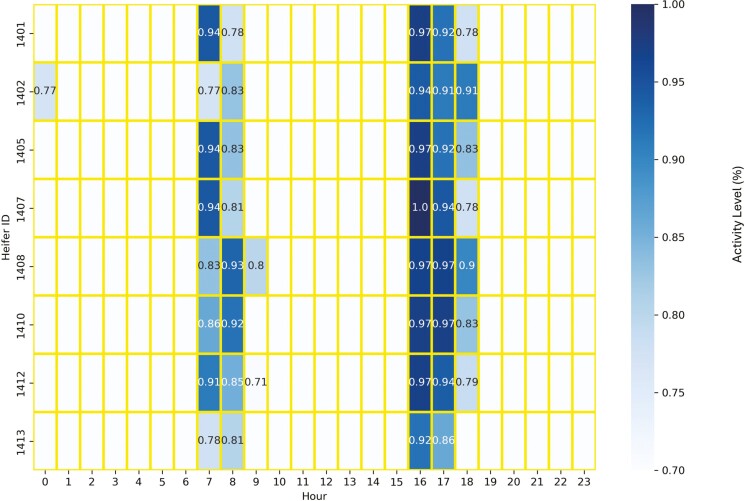
The active period of eight heifers based on movement variation (MV) during the study. High activity and associated MV levels are shown across the day (hours) for each heifer. The active periods were from 07:00–09:00 and from 16:00–19:00.

**Figure 2.**
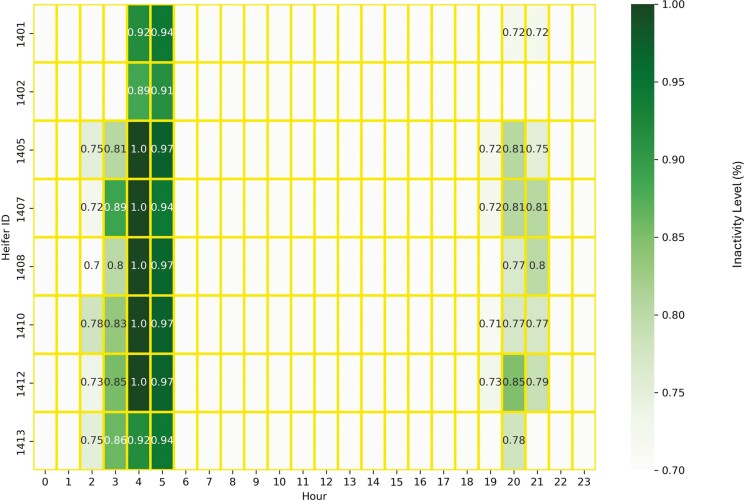
Inactive periods of eight heifers based on movement variation (MV) during the study. High inactivity and associated MV levels are shown across the day (hours) for each heifer. The inactive periods were 03:00–06:00 and 20:00–22:00.

The active period for eight heifers in Trial 1 spans from 07:00–09:00 and from 16:00–19:00 (Figure 1). Heifers were inactive from 03:00–06:00, and they were usually inactive from 20:00–22:00 (Figure 2). For our analyses, we defined two periods based on activity. Period $P_{1}$ spans 10 h containing two-time ranges from 06:00–11:00 and from 15:00–20:00, and period $P_{2}$ spans 13-h timeframe continuously from 06:00–19:00. The purpose of defining these two periods is to provide two time frames of differing length when cattle are typically more active than at night. The P_1_ period reflects the primary grazing bouts during the morning and evening (Gregorini, 2012). The P_2_ period includes daytime and some of the periods before sunrise and after sunset. The detection analyses were separately evaluated in these two time periods, $P_{1}$ and $P_{2}$.

#### Overview of proposed approaches.

**Figure 3.**
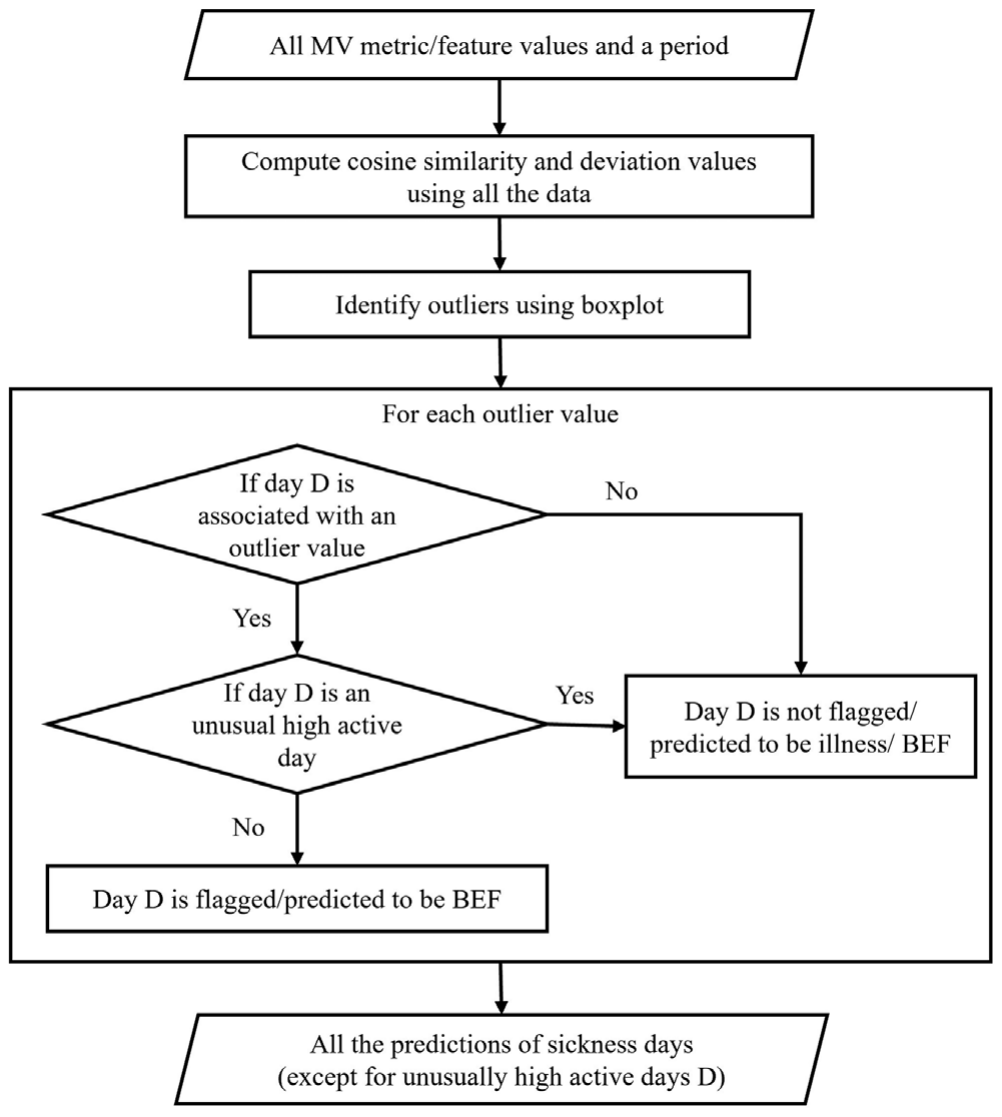
Flowchart/workflow of the developed data mining approaches.

Figure 3 illustrates the flowchart/workflow of the data mining approaches developed in this manuscript. Given all the MV metrics/features and a period, either $P_{1}$ or $P_{2}$, the CS and deviation values are computed in the “Pattern Mining - Cosine Similarity” and “Deviation Approach” sections, respectively. Next, a well-known statistical method, the boxplot, is applied to identify outliers in the CS and deviation values in the “Predicting sickness – Abnormally low activity” section. For each outlier, this indicates a change in behavior. If a day D is not associated with any outliers, it means that there is no flag or prediction for illness or BEF in our case. Otherwise, day D is further checked to see if it is an unusually high active day (as described in the “Noise Problem – Abnormally high activity” section). If it is an unusually high active day, it means that the day is not flagged or predicted to be illness. When day D satisfies both conditions, being associated with an outlier value and not being an unusually high active day, it is flagged or predicted to be BEF. The approach then outputs all the predictions of sickness days, except for unusually high active days D.

#### Pattern mining—cosine similarity.

A data instance consists of numerous features (1-h accelerometer metrics) organized in a chronological order over a designated time period P, which is represented as a vector. In our experiment, the period P, as defined in the “Active Periods” section, is either $P_{1}$ or $P_{2}$. Cosine Similarity is defined as the cosine of the angle between two data instances which determines their similarity. Given two data instances A and B, the equation of CS is as follows:


$$\begin{array}{l} similarity\left(A,B\right)=\;\frac{A\;\;\cdot \;B}{||A||*||\;B||} \end{array}$$

If similarity(A,B)=1, it implies that A and B are exactly the same. It means that the behavior of heifer is the same in these two periods. If similarity(A,B)=0, it implies that there are no similarities between A and B. It means that there is a change in behavior of heifer.

We considered four consecutive days, D-3, D-2, D-1 and D, and then computed the mean of CS among D and D-1, D and D-2, and D and D-3, This approach allowed us to evaluate the average similarity between day D and the previous 3 d.

#### Deviation approach.

Given a period of time P and a day D, the deviation between day D and its previous 3 d is described by two different equations as follows:


$$\begin{array}{l} sum\_deviation=\;\frac{\underset{i\;\in P}{\sum }\left|B_{i}^{D}-\bar{A_{i}}\right|}{C} \end{array}$$

or


$$\begin{array}{l} sum\_square\_deviation=\;\frac{\underset{i\;\in P}{\sum }{\left(|B_{i}^{D}-\;\bar{A_{i}}|\right)}^{2}}{C} \end{array}$$

where $B_{i}^{D}$ contains features at i -th hour on day D

C is the number of features in $\;B_{i}^{D}$

$\bar{A_{i}}=\;\frac{A_{i}^{D-1}+\;A_{i}^{D-2}+A_{i}^{D-3}}{3}$ in which $A_{i}^{D-1}$, $A_{i}^{D-2}$ and $A_{i}^{D-3}$ are features at i -th hour on day D-1, D-2 and D-3, respectively.

Intuitively, deviation calculates the difference in activity between today and the previous days during certain time of the day. This is based on our hypothesis that when a heifer becomes ill, it is likely to be less active during times it is normally active. If the value of sum_deviation or sum_square_deviation is high on a day D, it implies that there is an important change between day D and its three previous days, which implies the heifer became ill with BEF on day D. Otherwise, the heifer is considered healthy.

#### Predicting sickness—abnormally low activity.

After calculating CS and deviation values, to determine if a large deviation or a small CS should be considered abnormal behavior, the concept of boxplot outliers is used. A boxplot is a utilized to demonstrate the distribution of all cosine similarities or deviation values through the data quartiles based on the five criteria summarized below:

- Median: the middle value of CS values or deviation values
- First quartile (Q1): 25% of all CS values or deviation values fall below Q1.
- Third quartile (Q3): 75% of all CS values or deviation values fall below Q3.
- Min: Q1− 1.5∗(Q3−Q1)
- Max: Q3+1.5∗(Q3−Q1)

In the boxplot, a CS or deviation value x is an outlier if it is distant from other values. I f x<Min or x>Max, x was considered abnormal.

#### Noise problem—abnormally high activity.

Lastly, in our study, we needed to deal with data noise that occurred when a heifer was very active compared with the three previous days. In such cases, the deviation would be high, or the similarity was small even though the heifer was not ill and likely behaving normally. The abnormally high activity may be the result of cyclical changes in behavior such as estrus. Such noise can negatively affect the accuracy of predictions. To resolve this issue we defined a day as unusually active if during a period P on day D, |X| ≥0.8∗ |P| where $X=\;\{\;i\;\;|\;\;B_{i}^{D}-\;\bar{A_{i}}\;\geq 0\}$; |X| and |P| are the number of elements in the set X and instances in the period P, respectively. Set X consists of hour *i* that meets the given condition on day D. I.e., when the feature at hour *i* is greater than or equal to the average feature of the previous 3 d at the same hour, hour *i* is considered an unusually active hour and is put to *X.* When the unusually active hours in a day D (|*X*|) exceeds a percentage (0.8 * |*P*|), day D is considered unusually active day. Unusually high activity levels are not an expected symptom of BEF (Nandi and Negi, 1999). As a result, the usually high active days are not considered as illness when they are identified as outliers. However, all the data, including high active dates, were used in the previous computing CS and deviation values. The result of our approach addressing noise is shown in Table 2.

**Table 2.** The summary of dates with abnormally high activity for eight heifers in Trial 1. Bold and underlined dates reflect the two most common abnormally high activity dates, 2016-08-28 and 2016-09-04, respectively

Heifer ID	Dates (year/month/day) with abnormally high activity
1401	**2016-08-28**, 2016-09-04, 2016-09-18, 2016-09-30, 2016-10-01
1402	**2016-08-28**, 2016-09-04, 2016-09-15, 2016-09-16, 2016-09-18, 2016-09-27, 2016-09-30, 2016-10-04
1405	**2016-08-28**, 2016-09-04, 2016-09-30
1407	2016-09-04, 2016-09-18, 2016-09-30, 2016-10-01
1408	**2016-08-28**, 2016-10-01
1410	**2016-08-28**, 2016-09-03, 2016-09-04, 2016-09-30, 2016-10-01
1412	**2016-08-28**, 2016-09-04, 2016-09-18, 2016-09-30, 2016-10-01
1413	**2016-08-28**, 2016-09-02, 2016-09-03, 2016-09-04, 2016-10-04

## Results

### Bovine Ephemeral Fever

During the first trial in Rockhampton, heifers with identification numbers 1402 and 1413 were observed with BEF by the manager and verified by a veterinarian with a blood test on 12 September and 1 October 2016, respectively. Heifer 1402 was moved into a separate pen in the working facility and its accelerometer collar was removed and data downloaded after BEF detection, and put back in the pasture with the other heifers on 14 September 2016. Heifer 1413 was moved to the working facility on October 1 and the accelerometer collar remained on the animal until the end of the study. Heifer 1413 was returned to the pasture with the other heifers after BEF symptoms subsided.

At the Clermont study site (Trial 2), due to the short length of the available data of most cows, only four cows, O4, O2, Y3 and O7, are shown in the experiment. Three cows, O4, O2 and Y3 were first observed to get BEF on January 5, January 7, and January 21, 2022, respectively by manager and confirmed with a blood test. Cow O7 was reported sick from the manager on January 6^th^, 2022, but no blood test was conducted. Cow 07 became severely ill and was euthanized.

### Abnormally High Activity During Trial 1

Occasionally heifers in Trial 1 displayed abnormally high activity. August 28^th^, 2016 and September 4^th^, 2016 were the most common dates with abnormally high activity (Table 2), denoted in table with bold and underline text, respectively.

### Cosine Similarity and Deviation Analyses for the Trial 1

**Figure 4.**
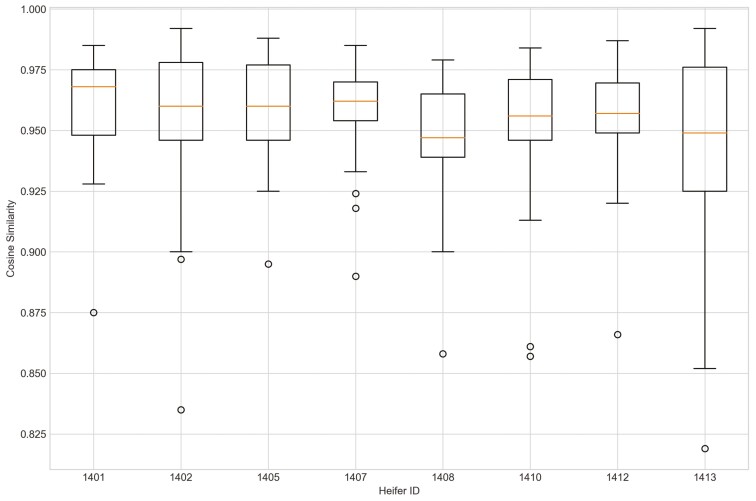
The outliers of eight heifers in Trial 1 during $P_{1}$ period when applying Cosine Similarity approach and utilizing preprocessed movement variation (MV).

**Figure 5.**
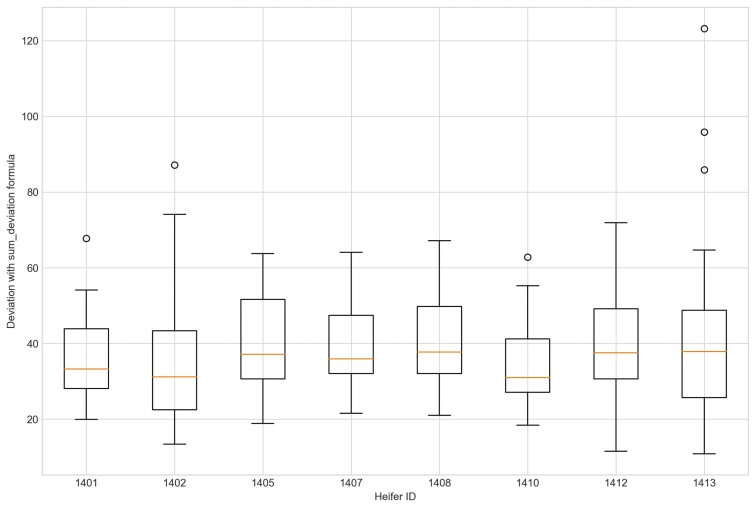
The outliers of eight heifers in Trial 1 during $P_{1}$ period when applying Deviation method with sum_deviation formula and utilizing preprocessed movement variation (MV).

**Figure 6.**
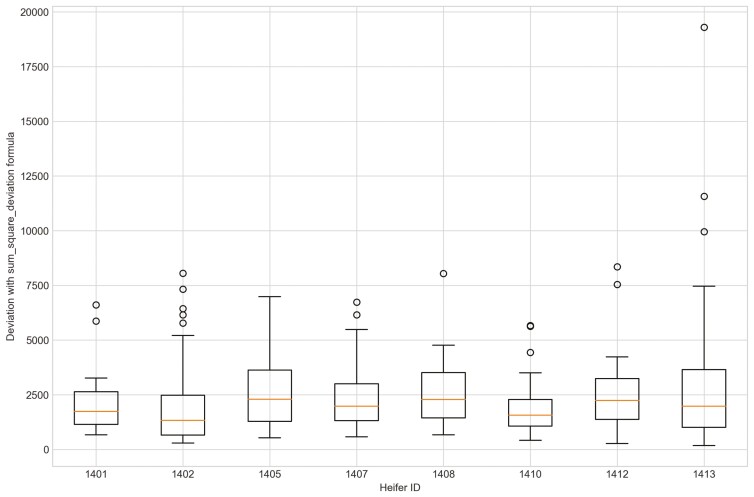
The outliers of eight heifers in Trial 1 during $P_{1}$ period when applying Deviation method with sum_square_deviation formula and utilizing preprocessed movement variation (MV).

Outliers from Trial 1 during $P_{1}$ period when applying CS were observed for all eight heifers (Figure 4). Four of the eight heifers had outliers using the sum_deviation formula during period $P_{1}$ (Figure 5), and most of the heifers had outliers with the sum_square_deviation formula (Figure 6). After removal of high activity days, the CS method identified abnormally low activity periods suggestive of illness for all eight heifers based on $P_{1}$ including the one of the two heifers that became ill with BEF (Table 3) on the exact date documented by a manager. The CS predicted unusual low activity (BEF prediction) for heifer 1402 the day before the manager observed BEF. Using the sums of squares formula and the $P_{1}$ period, seven of the eight heifers showed unusual low activity suggestive of prediction day, including the days the two heifers became ill with BEF. Heifers that showed unusual low activity, but were not observed with BEF are likely false positives. The deviation method using the sum_deviation formula and $P_{1}$ period correctly identified the days two of the eight heifers in Trial 1 were observed with BEF by the manager without any false positives.

**Table 3.** The summary of prediction dates for eights heifers in Trial 1 when applying Cosine Similarity approach, Deviation method with sum_deviation formula, and Deviation method with sum_square_deviation formula during $P_{1}$ period (from 06:00–11:00 and from 15:00–20:00). The bold text indicates the actual days those heifers with ID 1402 and 1413 got sick, as observed by the manager in comparison to the second column. Underlined dates reflect prediction dates within 3 d of the manager’s observation of illness. Italicized dates are likely false positives

Heifer ID	First BEF observation by manager (year/month/day)	Prediction dates (year/month/day)		
		Cosine Similarity	Deviation method with sum_deviation formula	Deviation method with sum_square_deviation formula
1401	None	*2016-09-19*	None	*2016-09-19*
1402	2016-09-12	2016-09-11, *2016-09-19*	**2016-09-12**	2016-09-11, **2016-09-12**, *2016-10-01, 2016-10-03*
1405	None	*2016-09-19*	None	None
1407	None	*2016-09-16, 2016-09-19, 2016-09-29*	None	*2016-09-19*
1408	None	*2016-09-19*	None	*2016-09-19*
1410	None	*2016-09-19, 2016-09-27*	None	*2016-09-19, 2016-09-23*
1412	None	*2016-09-19*	None	*2016-09-19*
1413	2016-10-01	**2016-10-01**	**2016-10-01** , 2016-10-02	**2016-10-01** , 2016-10-02

**Figure 7.**
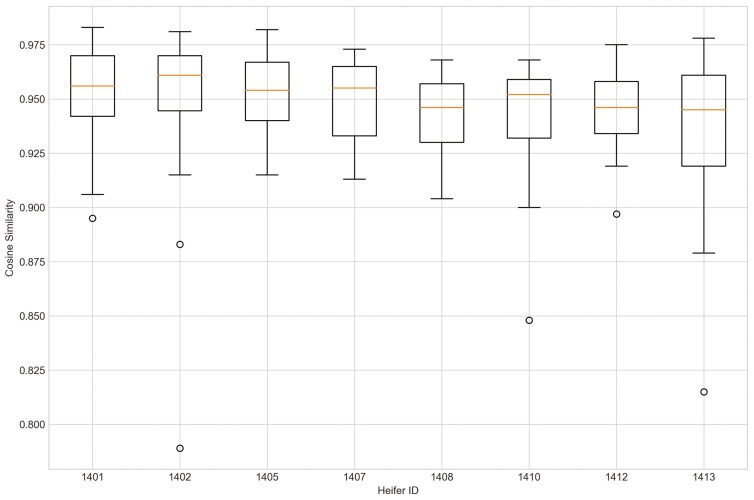
The outliers of eight heifers in Trial 1 during $P_{2}$ period when applying cosine similarity approach and utilizing preprocessed movement variation (MV).

**Figure 8.**
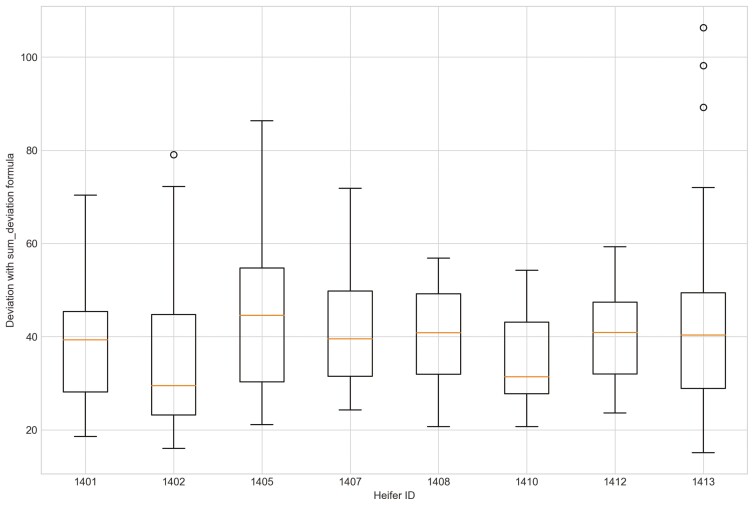
The outliers of eight heifers in Trial 1 during $P_{2}$ period when applying Deviation method with sum_deviation formula and utilizing preprocessed movement variation (MV).

**Figure 9.**
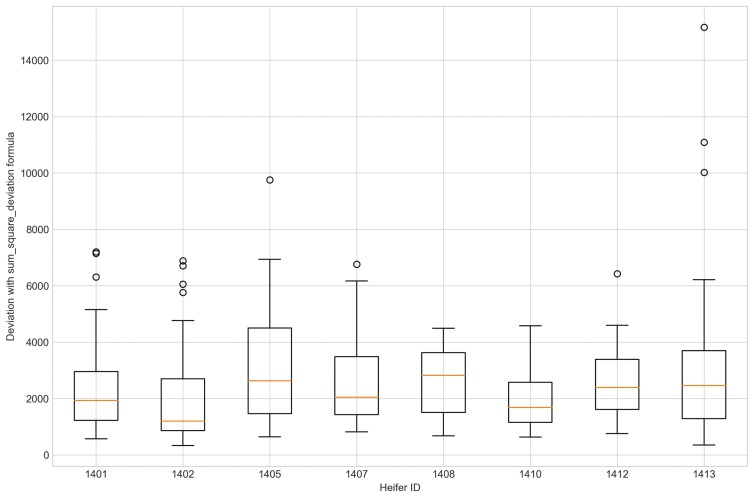
The outliers of eight heifers in Trial 1 during $P_{2}$ period when applying Deviation method with sum_square_deviation formula and utilizing preprocessed movement variation (MV).

**Figure 10.**
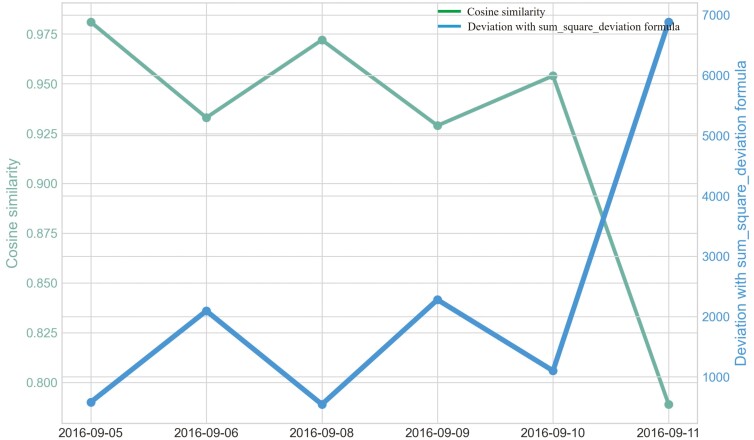
Changes in cosine similarity method and deviation method using sum_square_deviation formula for heifer 1402 prior to and during the onset of bovine ephemeral fever (September 5^th^, 2016 to the prediction day, September 11^th^, 2016 during Trial 1) using $P_{2}$ period.

**Figure 11.**
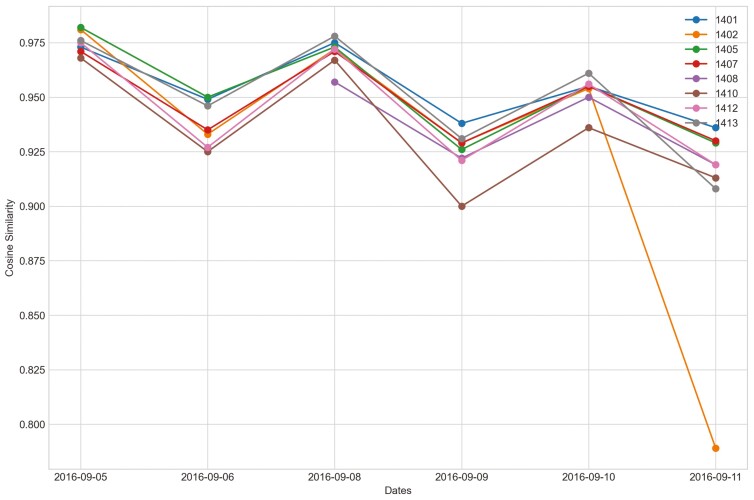
Changes in the cosine similarity method for all heifers from September 5^th^, 2016 to the prediction day of heifer 1402, September 11^th^, 2016 in Trial 1 during $P_{2}$ period.

**Figure 12.**
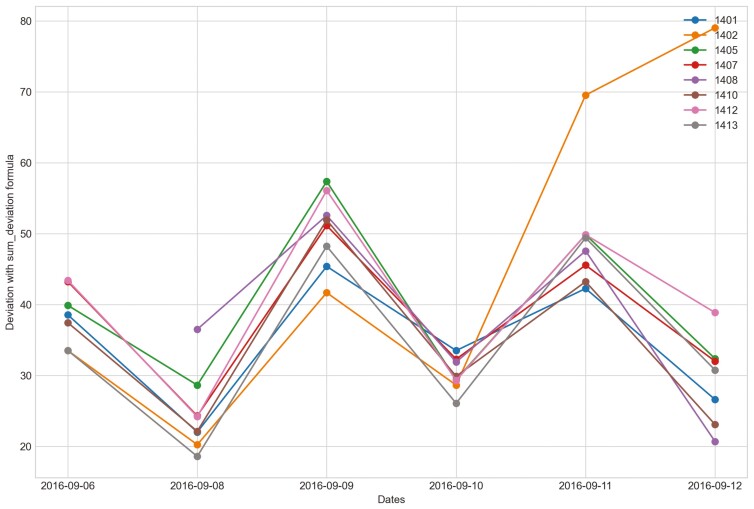
Changes in the deviation method utilizing sum_deviation for all heifers from September 6^th^, 2016 to the prediction day of heifer 1402 September 12^th^, 2016 in Trial 1 during $P_{2}$ period.

**Figure 13.**
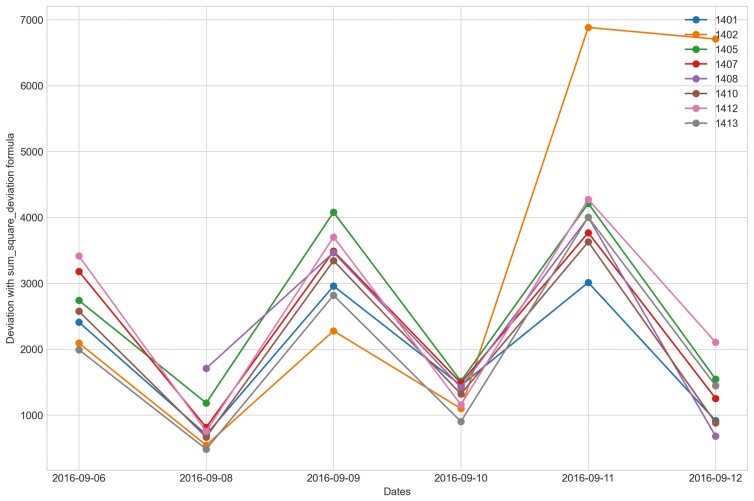
Changes in the deviation method using sum_square_deviation formula for all heifers from September 6^th^, 2016 to September 12^th^, 2016 in Trial 1 during $P_{2}$ period.

**Figure 14.**
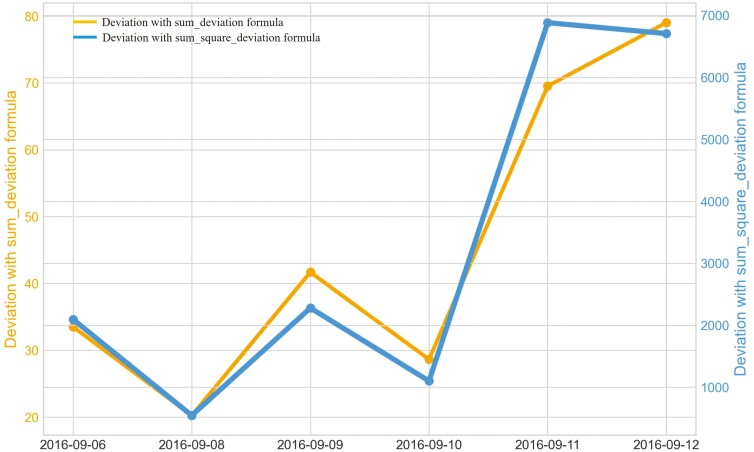
Changes in the deviation method utilizing sum_deviation and sum_square_deviation formulas for heifer 1402 before and during onset of bovine ephemeral fever (September 6^th^, 2016 to the another prediction day, September 12^th^, 2016 during Trial 1) using $P_{2}$ period.

**Figure 15.**
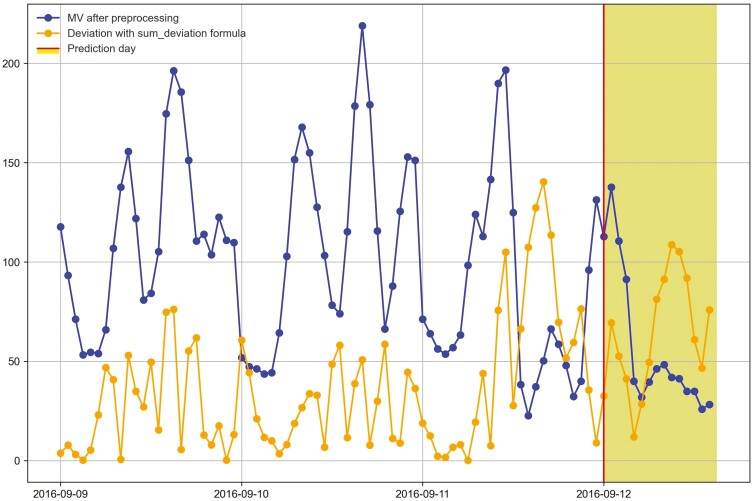
Movement variation (MV) metric and the hourly deviation value using sum_deviation formula of heifer 1402 during period 2 within 3 d before bovine ephemeral fever was predicted during Trial 1 (September 12^th^, 2016).

**Figure 16.**
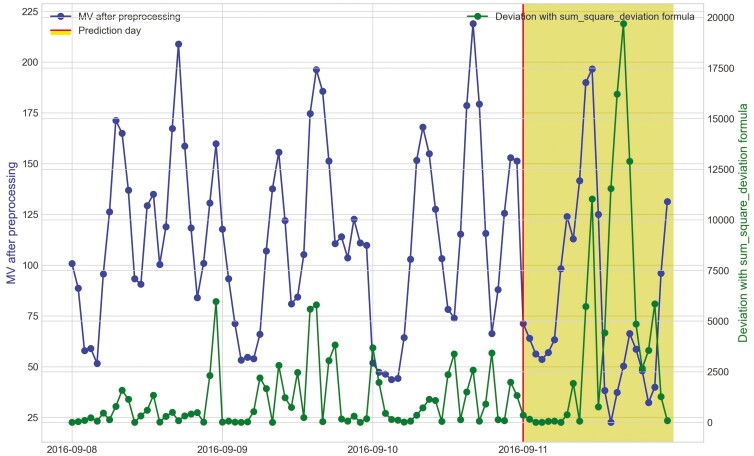
Movement variation (MV) metric of period 2 and the hourly deviation value using sum_square_deviation formula of heifer 1402 within 3 d before bovine ephemeral fever was predicted on September 11^th^, 2016 during Trial 1.

**Figure 17.**
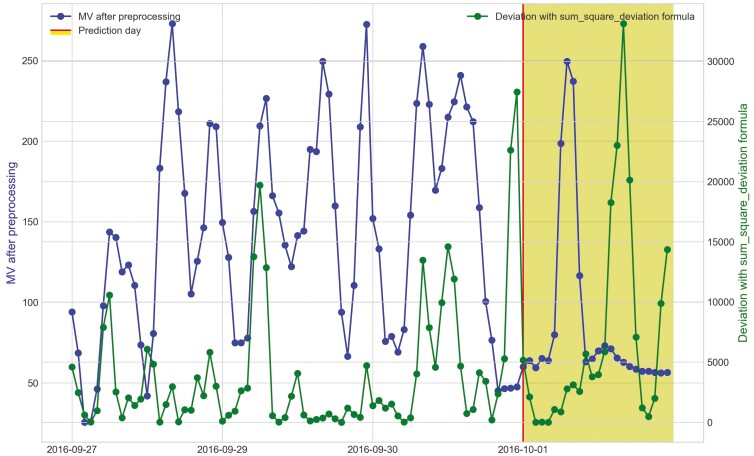
Movement variation (MV) metric of period 2 and the hourly deviation value using sum_square_deviation formula of heifer 1413 during Trial 1 within 3 d before its prediction day, October 1^st^, 2016.

Outliers were identified in five of eight heifers during $P_{2}$ period when applying CS (Figure 7). Only two of eight heifers had outliers for period P_2_ for the sum_deviation formula, and these heifers became ill with BEF (Figure 8). Outliers were found for six of eight heifers using the sum_square_deviation formula and period $P_{2}$ (Figure 9). After removing the abnormally high activity days, the CS method predicted abnormally low activity for five of eight heifers. Heifer 1402 was predicted to have BEF one day before manager observed it using the CS method (Figures 10 and 11). One of the prediction days with CS was when the manager observed BEF for heifer 1413 (Table 4). The two heifers observed and diagnosed with BEF, were detected using the deviation method with sum_deviation formula during $period\;P_{2}$.With the square deviation method, illness was predicted for three of the eight heifers including the two heifers that were observed with BEF (Table 4). Similar to the CS method, the deviation approach with sum_square_deviation formula predicted BEF for heifer 1402 one day earlier than the manager, while the deviation approach with sum_deviation formula predicted it on the same day (Figures 12–16). Deviation with both formulas provided predictions that matched with the manager’s observation for heifer 1413 (Figure 17). As with $P_{1}$, heifers that were detected with low activity, but were not observed with BEF symptoms are likely false positives.

**Table 4.** The summary of prediction dates for eights heifers in Trial 1when applying Deviation method with sum_square_deviation formula during $P_{2}$ period (from 06:00–19:00). The bold text indicates the actual days those heifers with ID 1402 and 1413 got sick, as observed by the manager in comparison to the second column. Underlined dates reflect prediction dates within 3 d of the manager’s observation of illness. Italicized dates are likely false positives

Heifer ID	First BEF observation by manager (year/month/day)	Prediction dates (year/month/day)		
		Cosine Similarity	Deviation method with sum_deviation formula	Deviation method with sum_square_deviation formula
1401	None	*2016-10-03*	None	*2016-10-03*
1402	2016-09-12	2016-09-11	**2016-09-12**	2016-09-11, **2016-09-12**, *2016-10-03*
1405	None	None	None	None
1407	None	None	None	None
1408	None	None	None	None
1410	None	*2016-09-27*	None	None
1412	None	*2016-09-19*	None	None
1413	2016-10-01	**2016-10-01**	**2016-10-01** , 2016-10-02	**2016-10-01** , 2016-10-02

### Cosine Similarity and Deviation Analysis for Trial 2

In Trial 2, four cows, O4, O2, Y3 and O7 were first observed to get BEF on January 5^th^ 2022, January 7^th^ 2022, December 23^st^ 2021, and January 6^th^ 2022, respectively. Three cows, O4, O2 and Y3 were confirm with blood tests. Similar to Trial 1, after removing the abnormally high activity days, CS accurately identified cow Y3’s sickness on the same day as it was noticed by the manager. A change in cow O2’s behavior (illness detection) was identified one day earlier than the manager’s observation of BEF. Cosine similarity method did not succeed in predicting the sickness of cow O4 and O7. During $P_{1}$ period, the deviation method, using both formulas (squared and not squared), successfully identified the day when cow Y3 became ill and was observed and diagnosed by the manager. Cow O2 displayed behavioral change one day prior to the manager’s diagnosis using the deviation method. The deviation (both formulas) identified behavioral changes in cow O4 up to 5 d before the manager observed BEF. Cow O7 showed alterations in its behavior 4 d before the manager’s observation using the deviation method. (Table 5).

**Table 5.** The summary of prediction dates for three cows in Trial 2 when applying Cosine Similarity approach, Deviation method with sum_deviation formula, and Deviation method with sum_square_deviation formula during $P_{1}$ period (from 06:00–11:00 and from 15:00–20:00). The bold text indicates the actual days those cows got sick, as observed by the manager in comparison to the second column. Underlined dates reflect prediction dates within 3 d of the manager’s observation of illness. Italicized dates are likely false positives. Dates that are not underlined, bold or italicized may or may not be false positives

Cow ID	First BEF observation by manager (year/month/day)	Prediction dates (year/month/day)		
		Cosine similarity	Deviation method with sum_deviation formula	Deviation method with sum_square_deviation formula
O4	2022-01-05	None	2021-12-31	2021-12-31, *2022-02-28, 2022-03-04*
O2	2022-01-07	2022-01-06, 2022-01-08, *2022-01-14, 2022-02-13*	2022-01-06	2022-01-06, *2022-02-10, 2022-02-13, 2022-02-23*
Y3	2021-12-23	2021-12-22, **2021-12-23**, 2021-12-24	2021-12-21, 2021-12-22, **2021-12-23**	*2021-12-16,* 2021-12-19, 2021-12-21, 2021-12-22, **2021-12-23**
O7	2022-01-06	2022-01-08, 2022-01-09, 2022-01-10, 2022-01-11, 2022-01-12, *2022-02-04, 2022-02-05*	2022-01-02, 2022-01-09	2022-01-02, 2022-01-08, 2022-01-09

**Figure 18.**
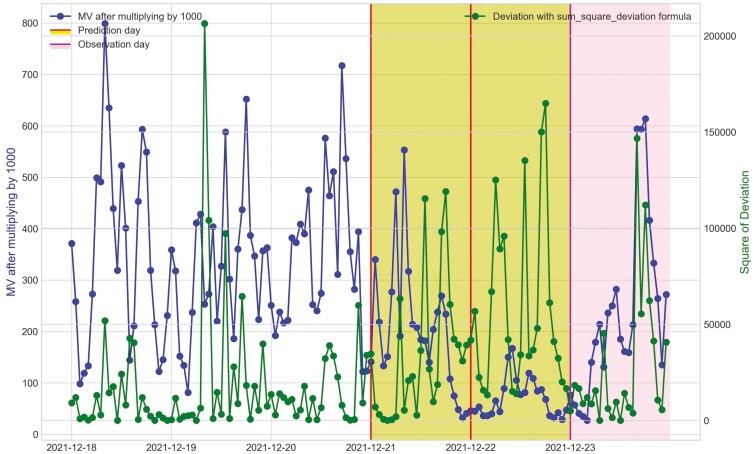
Movement variation (MV) metric and the hourly deviation value using sum_square_deviation formula of cow Y3 in Trial 2 during $P_{2}$ period with 3 d before its prediction day, December 21^st^, 2021 and December 22^nd^, 2021 and its observation day as BEF, December 23^rd^, 2021.

**Figure 19.**
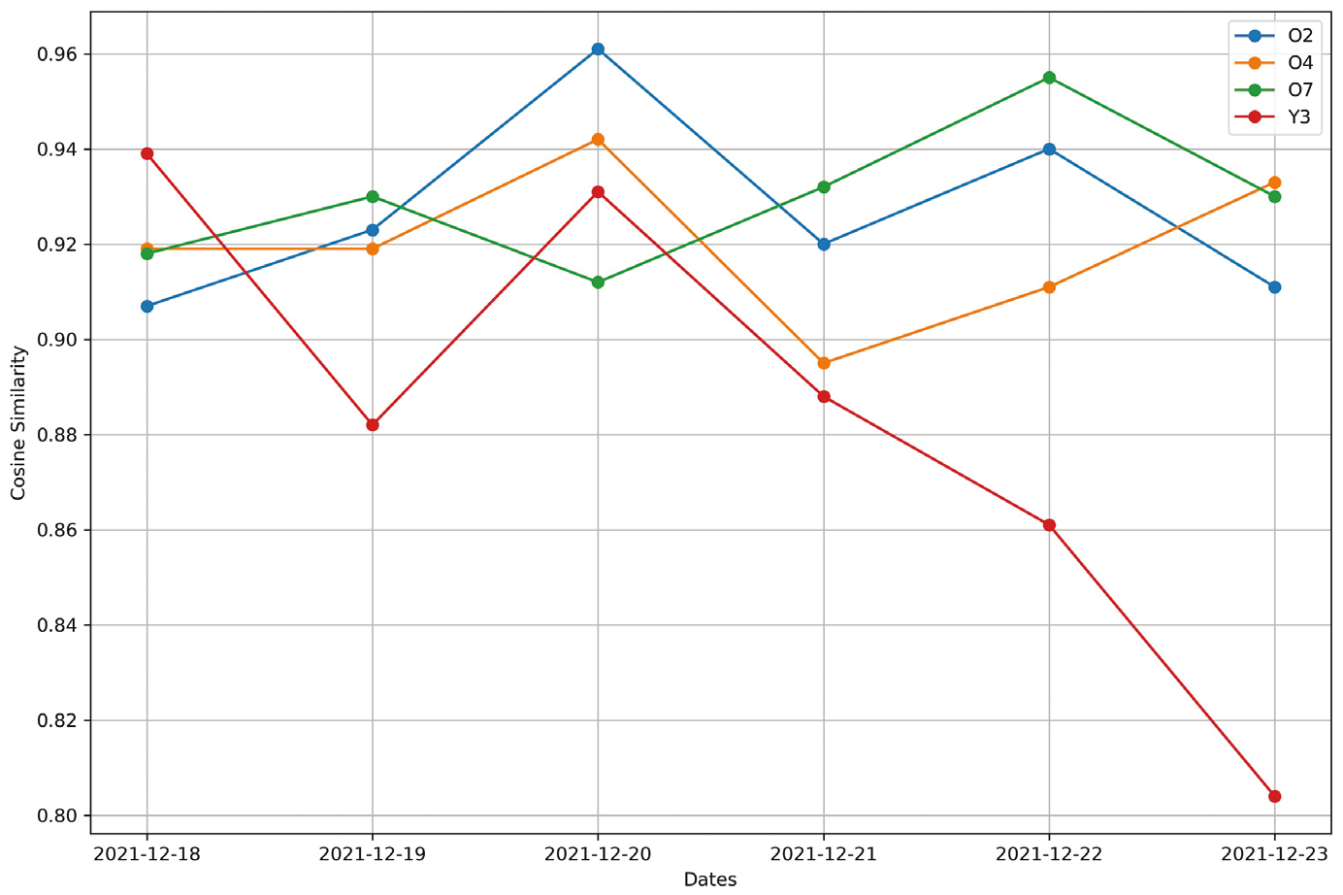
Changes in the cosine similarity method for all cows from December 18^th^, 2021 to December 23^rd^, 2021 in Trial 2 during $P_{2}$ period.

**Figure 20.**
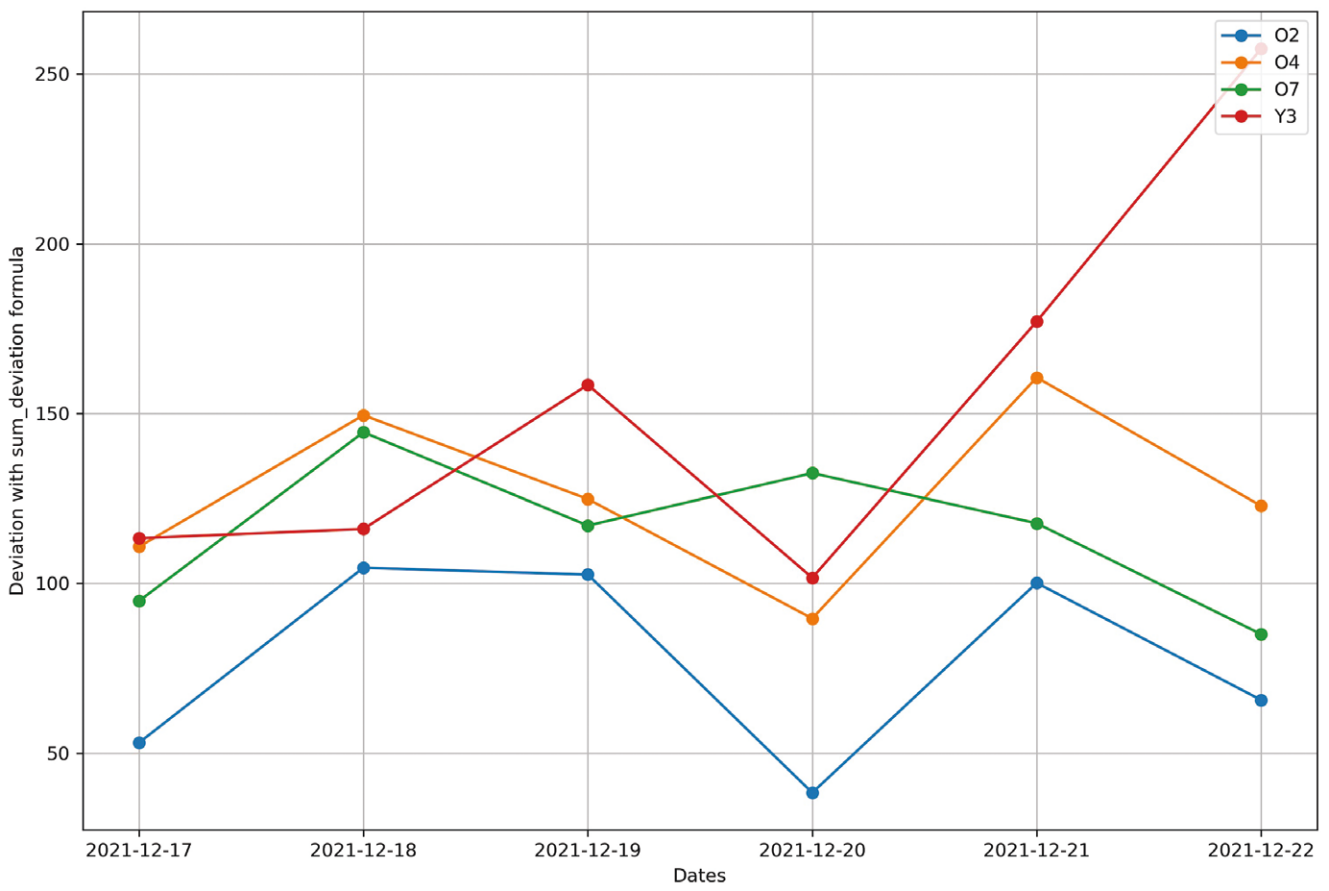
Changes in the deviation method using sum_deviation formula for all cows from December 17^th^, 2021 to December 22^nd^, 2021 in Trial 2 during $P_{2}$ period.

**Figure 21.**
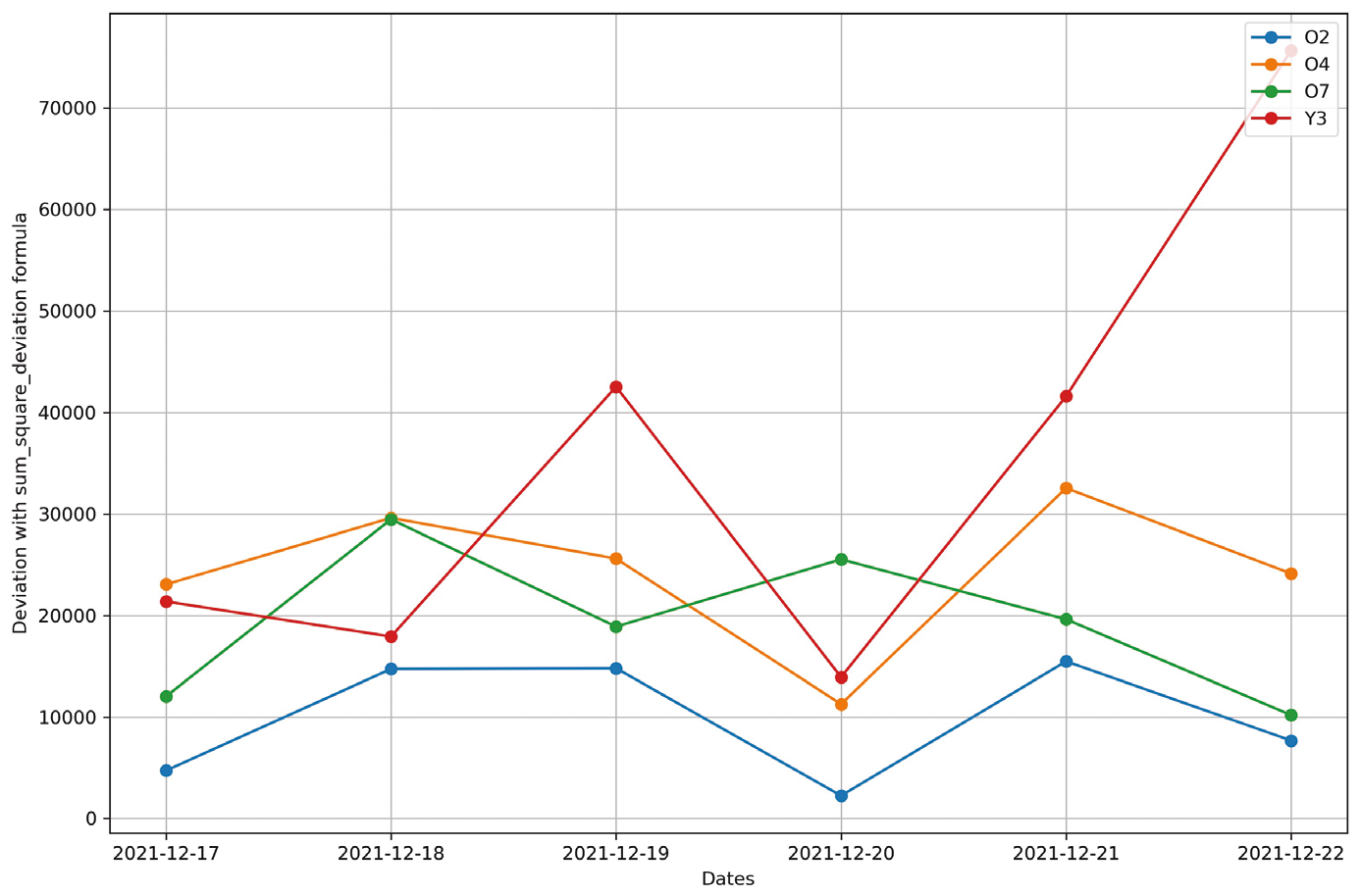
Changes in the deviation method using sum_square_deviation formula for all heifers from September 6^th^, 2016 to September 12^th^, 2016 in Trial 1 during $P_{2}$ period.

**Figure 22.**
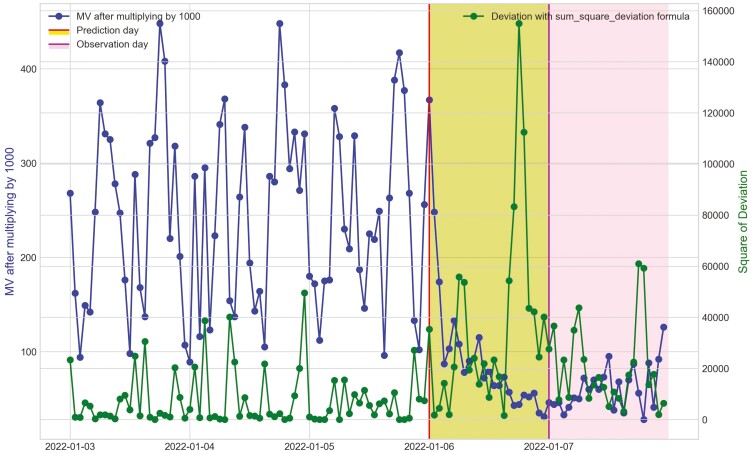
Movement variation (MV) metric and the hourly deviation value using sum_square_deviation formula of cow O2 in Trial 2 during $P_{2}$ period with 3 d before its prediction day, January 6^th^, 2022 and its observation day as BEF, January 7^th^, 2022.

**Figure 23.**
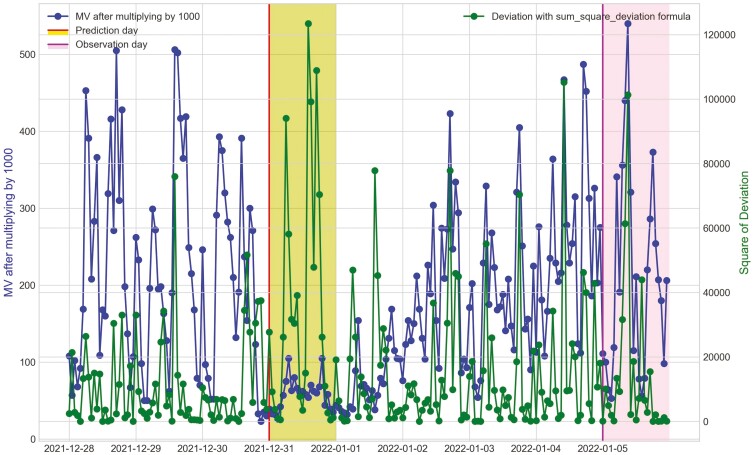
Movement variation (MV) metric and the hourly deviation value using sum_square_deviation formula of cow O4 in Trial 2 during $P_{2}$ period with 3 d before its prediction day, December 31^st^, 2021 and its observation day as BEF, January 5^th^, 2022.

**Figure 24.**
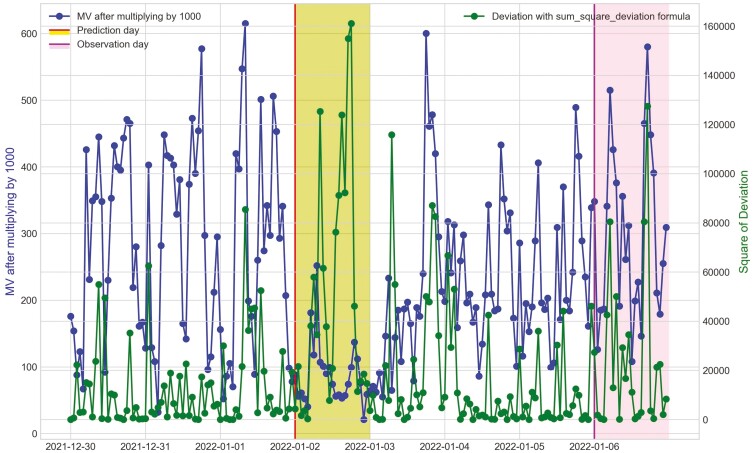
Movement variation (MV) metric and the hourly deviation value using sum_square_deviation formula of cow O7 in Trial 2 during $P_{2}$ period with 3 d before its prediction day, January 2^nd^, 2022 and yellow area and its observation day as BEF, January 6^th^, 2022.

During period $P_{2}$, both the CS method and the deviation (using both formulas) successfully detected unusual changes in activity for cow Y3 (Figures 18–21). The CS method detected the changes in behavior in cow O4 on the same day as manager but failed to predict illness of cows O2 and O7. For cow O2, the deviation method using sum_deviation formula did not effectively predict the occurrence of illness, while the sum_square_deviation formula detected behavior changes one day before the manager (Figure 22). Similar to period $P_{1}$, the deviation (both formulas) identified changes in behavior of cows O4 (Figure 23) and O7 (Figure 24) five and 4 d before the manager observed BEF, respectively (Table 6).

**Table 6.** The summary of prediction dates for three cows in Trial 2 when applying Cosine Similarity approach, Deviation method with sum_deviation formula, and Deviation method with sum_square_deviation formula during $P_{2}$ period (from 06:00–19:00). The bold text indicates the actual days those cows got sick, as observed by the manager in comparison to the second column. Underlined dates reflect prediction dates within 3 d of the manager’s observation of illness. Italicized dates are likely false positives. Dates that are not underlined, bold or italicized may or may not be false positives

Cow ID	First BEF observation by manager (year/month/day)	Prediction dates (year/month/day)		
		Cosine similarity	Deviation method with sum_deviation formula	Deviation method with sum_square_deviation formula
O4	2021-01-05	**2022-01-05**	2021-12-31	2021-12-31, *2022-02-28, 2022-03-01, 2022-03-03, 2022-03-04*
O2	2022-01-07	*2022-01-14, 2022-01-15*	*2022-02-10*	2022-01-06, *2022-02-08, 2022-02-10, 2022-02-11, 2022-02-13, 2022-02-23, 2022-02-25, 2022-02-27*
Y3	2021-12-23	**2021-12-23**	2021-12-21, 2021-12-22	*2021-12-16*, 2021-12-19, 2021-12-21, 2021-12-22
O7	2022-01-06	2022-01-09, 2022-01-10, 2022-01-11, 2022-01-12, *2022-02-04, 2022-02-05*	2022-01-02	2022-01-02, 2022-01-09

### Evaluation of Results with Available Ground Truth Data and All Data

Notably, the pattern mining methods in our manuscript belong to unsupervised learning, the evaluation does not rely on metrics like sensitivity and specificity, which are specific to supervised learning, although they can be utilized in unsupervised learning when ground truth is available. Considering the equations of sensitivity and specificity as follows:


$$\begin{array}{l} sensitivity=\frac{TP}{TP+FN} \end{array}$$

$$\begin{array}{l} specif\;\;icity=\frac{TN}{TN+FP} \end{array}$$

In the above equations, true positive (TP) is the number of animals which are correctly classified as BEF by the method; false positive (FP) is the number of animals which are incorrectly classified as BEF by the method; true negative (TN) is the number of animals which are healthy and correctly classified as healthy by the method; false negative (FN) is the number of animals which has BEF but incorrectly classified as healthy by the methods.

Ground truth data is a necessity to calculate these two metrics. However, in our study, the ground truth, blood tests verified BEF, is not available for all the animals in both Trials 1 and 2. As a result, we are unable to report the sensitivity and specificity based on all of our data. Therefore, the values of sensitivity and specificity are reported below, based on the available ground truth (animals with blood tests). We considered the combined results from three methods over two periods to determine the metrics. Specifically, an animal is classified as correctly predicted as BEF if it is identified as ill within 3 d of the manager’s observation by one of the methods during either period.

- Trial 1 has ground truth for two heifers 1402 and 1413. Both are diagnosed with BEF, and there are no healthy animals in this ground truth data set. From Tables 3 and 4, both heifers are correctly predicted their sickness, thus TP=2, FP=0, TN=0, FN=0. We have sensitivity=1 and specificity is undefined.
- Trial 2 has ground truth for three cows O4, O2 and Y3. Similar to Trial 1, the specificity is undefined and sensitivity=1 based on Tables 5 and 6.

For our entire dataset in Trials 1 and 2, under the field of pattern mining in data mining, the evaluation includes both objective and subjective measures, which are presented in the above sections through all tables and figures as follows:

- The *objective measure* is based on the facts that the animal was diagnosed with BEF by the manger’s observation and blood tests. The objective values are the days on the second column of Tables 3–6. Because the incubation period is typically 2–4 d in most cases, the objective value in this manuscript is within 3 d of the BEF diagnosis, which is reflected by the underlined dates in Tables 3–6.
- The *subjective measure* is based on the analysis of patterns by users. In our manuscripts, this includes the visual inspection by experts or humans via Figures 4–24, which show individual and group animal data. For figures representing individual animals, the predicted days indicate a decrease in the raw data, suggesting that the animal is abnormally low activity. This decrease is a typical clinical sign of BEF, where the animals may exhibit lameness, lie down and refuse to move. For figures representing groups of animals in each trial, when an animal is predicted to be sick, there is a noticeable change in behavior, either an increase in deviation or a decrease in CS.

## DISCUSSION

In this study, two methods applied to two different trials were able to detect behavioral changes that occurred as cattle became ill with BEF. False positive predictions may arise from various factors such as weather conditions, abnormal weather events, cyclic shifts in behavior, or other comparable diseases. Despite the presence of false positive results, we were successful in identifying when heifers in Trial 1 and cows in Trial 2 became ill with BEF, and even earlier than the manager. When the algorithms detected an illness the day before or on the day following previous detection in Trial 1, we do not consider it a false positive since we are unable to know exactly when the animal became sick. We only know when the manager observed the animal being sick. In Trial 2, the pasture was more extensive and on a commercial operation rather than a research farm. The manager may not have noticed the animal when it first became ill. Correspondingly, detection a few days before the manager observed the animal being ill may not be a false positive.

Use of $P_{2}$ period led to more false positives than those that used $P_{1}$ period with both the CS and Deviation methods in Trial 1. One potential explanation for this is the duration of the $P_{2}$ period is longer than $P_{1}$ period and reflects more of the animal’s daily behavioral pattern. Midday (11:00–15:00), excluded in P_1_, includes inactive periods that may affect the algorithms and sometime make detection more difficult. When cattle are usually inactive, it is improbable for an accelerometer to detect a behavioral change when they become ill, because a decrease in activity is not noticeable.

When cattle become ill, we expected a decline in CS and an increase in deviation because the animal becomes less active. For example, there was a clear decrease in CS and a notable increase in the sum_square_deviation formula when heifer 1402 became ill (Figure 10). Figure 11 illustrates the change in CS for heifers in Trial 1 during $P_{2}$ period, prior to the prediction date of heifer 1402. It is evident that there is a significant decrease in CS for heifer 1402, while the other heifers do not. Figures 12 and 13 shows the change deviation value using sum_deviation and sum_square_deviation formulas in Trial 1 during $P_{2}$ period, respectively, prior to the prediction date of heifer 1402. Once again, the deviation of heifer 1402 was distinct from other heifers, displaying a significant increase. This demonstrates that there was a remarkable change in behavior associated with the onset of BEF. Moreover, the deviation value, computed with sum_square_deviation formula, increases more rapidly than when using sum_deviation formula, potentially leading to earlier detection in some cases (Figure 14). On September 11^th^, 2016, an increase can be observed with the sum_deviation formula. However, it is not as large of change compared to other days until September 12^th^, 2016. Note that on September 12^th^, 2016, data ends at 15:00 for heifer 1402, because the manager removed its collar to help reduce symptoms associated with BEF.

The processed raw data from the accelerometer, movement variation, shows the diurnal changes in behavior and the distinct decline in activity when heifers in Trial 1 became ill (Figures 15 and 16). The hourly deviation values using sum_deviation and sum_square_deviation formulas identify the behavior changes as shown for heifer 1402 in Figures 15 and 16 on September 12, 2016 and September 11, 2016, respectively. Notably, although heifer 1402 had missing data in the afternoon (after 3:00 p.m.) on September 12, 2016, Figure 15 illustrates that the available data reveals a downward trend in MV values, which results in an upward trend in deviation with sum_deviation values. This pattern suggests a potential for illness prediction for heifer 1402 based on the available data and our methods. The sum_square_deviation formula provided a more noticeable distinction when the heifer became ill one day earlier, highlighting a hourly significant gap between deviation value and MV metric compared to the sum_deviation formula. Like heifer 1402, heifer 1413 showed a decrease in activity as it became ill (Figure 17). However, there was later an increase in MV and activity as the heifer was trailed to the pens for treatment. This change from inactivity to movement during trailing was noted by the deviation method. Stiffness and changes in walking gait are often symptoms of BEF (Nandi and Negi, 1999).

For Trial 2, similar to Trial 1, Figure 18 shows that the processed raw accelerometer data, movement variation, decreased on the predicted days for cow Y3. Figures 19–21 illustrate the pattern, with a decline in CS and an increase in deviation during the period when the animal was identified as sick. Figure 19 displays the change in CS for four cows in Trial 2 during $P_{2}$ period, prior to the prediction date for cow Y3. It is clear that cow Y3 experienced a significant decline in CS, while the other cows did not. Figures 20 and 21 show the changes in deviation values, calculated using sum_deviation and sum_square_deviation formulas, respectively, during $P_{2}$ period, prior to the prediction dates of cow Y3. Once again, cow Y3’s deviation was markedly different from that of other cows, with a significant increase. Similarly, the use of sum_square_deviation formula resulted in a notable increase in deviation before cows were observed to be ill (Figures 22–24). Movement variation decreased during normal active periods (e.g., $P_{1}$ and $P_{2}$) on the prediction day and for at least one day following the prediction (Figures 22–24). This decrease in MV suggests a decrease in activity, possibly from the onset of BEF. Predictions of BEF occurred earlier than observation of BEF by the manager. Cow O4 showed changes in behavior 5 d before manager recognized its sickness, but the early detection may not be related to BEF, as this disease often lasts for 3 d. However, MV increased to levels during the later part of the interim between prediction and the manager’s observation of BEF, which is an indication of increased activity to levels a day or two after prediction of BEF (Figure 23). Cow O7 displayed behavioral changes 4 d prior to the manager’s observation. Although we cannot be sure, this may be a detection of an initial stages of BEF development. Similar to cow O4, cow 07 showed low MV levels the day of and day after BEF detection and then increased to levels recorded before BEF detection prior to observation and diagnosis by the manager (Figure 24). Although we cannot be sure that the algorithm correctly detected the onset of BEF for all the cows in Trial 2, the identification of behavioral changes prior to illness appears promising.

Although CS is a well-known method in data mining (Tan et al., 2016), our proposed method (deviation) outperforms it by generating fewer false positives and predicting either on the same day or even earlier than the manager. The deviation method computed with the sum_square_deviation formula might be the preferred choice for prediction among the methods, primarily due to its rapid increase and subsequent ability to forecast behavior changes sooner. However, the deviation method with sum_deviation formula had fewer false positive, which would make it preferable if false positives are a major concern. Tobin et al. (2020) demonstrated that the accelerometers have potential to detect the distinct movement patterns in heifer when they became sick. They found clear changes in accelerometer readings (movement intensity) as the heifers became ill with BEF. However, they did not develop a detection algorithm. In this paper using data from Tobin et al. (2020) and additional data (Trial 2), we described the development and implementation of two algorithms to identify changes in cattle behavior associated with illness. The deviation method using the shorter P_1_ period has potential for identifying illnesses such as BEF. Our methods have potential to detect illness sooner than managers observe it (e.g., cow Y3 in Trial 2).

Finally, our methods displayed promise in the identification of various diseases using commercial real-time sensor technologies, supported by the identification of behavioral changes in animals when they become ill. This approach was founded on the hypothesis that when animals are unwell, they tend to be less active during the times when they are expected to be active. For instance, a study conducted by Gurule et al. (2022) examined the impact of mycotoxin consumption in ewes. In this study, some ewes that consumed mold-contaminated feed with mycotoxins, had difficulty walking and reduced their feed intake. These ewes appeared depressed and less active. Gurule et al. (2022) found that HerdDogg biometric accelerometers (https://www.herddogg.com/) detected the activity changes. HerdDogg accelerometers are a commercial near-real time sensor that could be used for remote monitoring of livestock health.

Currently, the limited battery life of the accelerometers used in this study (3 mo or less) limits their potential use in extensive rangeland pastures. Managers cannot move cattle to working facilities to replace batteries every few months when grazing extensive pastures. However, ongoing technological improvements, including placing solar panels on the devices, may make accelerometers and other on-animal sensors more practical for rangeland cattle operations in the future.

## CONCLUSION

In this study, we utilized tri-axial accelerometer data to develop algorithms to detect changes in behavior patterns when heifers and cows became sick with bovine ephemeral fever. By applying two distinct methodologies, CS and deviation from previous behavioral patterns, we are able to detect when the heifers and cows got sick on the same day as the manager, or even earlier. The deviation method outperformed the CS method in both trials by successfully predicting the majority of sick heifers and cows, while also producing fewer false positives in Trial 1 and 2. Although more research is needed, our approaches have potential to use with real-time accelerometers sensing equipment to help managers remotely monitor cattle health and identify when animals should be checked and possibly treated for illness while grazing pastures. Further research and continued algorithm development may minimize false positives, reduce false negatives and improve illness detection. Our approaches rely on the identification of a decrease in activity as the animal becomes ill. Diseases where reduced activity would not be a consequence of expected symptoms will likely require different methods and detection algorithms.
